# A Box–Behnken design–assisted RP-HPLC method development and optimization for simultaneous determination of four antiviral drugs in pharmaceutical dosage forms

**DOI:** 10.1038/s41598-026-50549-4

**Published:** 2026-05-11

**Authors:** Fatma H. Ghazy, Ramzia I. El-Bagary, Samar H. Fahim, Sally T. Mahmoud

**Affiliations:** 1 Pharmaceutical Chemistry Department, Faculty of Pharmacy, Cairo University, Kasr El-Aini St., P.O.11562, Cairo, Egypt; 2 Pharmaceutical Chemistry Department, School of Pharmacy, Km 22 Cairo-Alexandria Desert Road, New Giza, Cairo Egypt

**Keywords:** RP-HPLC, Quality by design (QbD), Box–Behnken design (BBD), COVID-19, Chemistry, Drug discovery, Health care

## Abstract

The advent of COVID-19 has highlighted the value of repurposing existing antiviral medications and the importance of developing efficient analytical methods to assist their quality control. Simultaneous determination of multiple agents saves time and resources in pharmaceutical analysis. Moreover, the analytical demand during health emergencies underscores the need for validated, rapid, and reliable methods that can support both therapeutic evaluation and regulatory decisions. In this study, we developed and optimized a reversed-phase high-performance liquid chromatography (RP-HPLC) method to measure four repurposed antiviral drugs: favipiravir, sofosbuvir, ledipasvir, and remdesivir. A Box–Behnken design (BBD) approach was employed to study how buffer pH, the amount of organic solvent in the mobile phase, and flow rate affect key outcomes like resolution and run time. The separation was done on INERTSIL ODS-3 C18 column (5 μm, 250 × 4.6 mm) under isocratic elution conditions using acetonitrile (50:50, v/v) and ammonium formate buffer (0.02 M, adjusted to pH 2.8 with formic acid) as the mobile phase at flow rate 1 mL/min. Detection was performed at 240 nm. The method demonstrated good system suitability and met ICH Q2 (R2) validation standards, exhibiting satisfactory linearity (R² > 0.999), accuracy (recoveries within 98–102%), and precision (RSD < 2%). The sustainability of the proposed method was comprehensively evaluated using AGREE, MoGAPI, BAGI, and RAPI tools to assess its greenness, blueness, and whiteness. The proposed method offers a practical and efficient analytical platform for quality control laboratories, enabling reliable routine analysis of multiple antiviral agents in pharmaceutical products, particularly for post-pandemic preparedness and therapeutic surveillance.

## Introduction

Viruses represent the primary agents responsible for morbidity and mortality globally, and they have a devastating impact on the human population^1^. The basic components of a virus are its nucleic acid, which may be either single-stranded or double-stranded (DNA or RNA) enclosed with a protein shell known as capsid; some viruses also have an external envelope made of lipids and proteins. Viruses replicate by using the host’s cellular systems, making them obligatory intracellular infections. The development of medications with specific toxicity against viruses is hampered by these features^2^. Based on their genetic makeup, viruses can be divided into three groups: reverse transcription viruses which include the human immunodeficiency virus (HIV), RNA viruses such as hepatitis C, and influenza viruses, and DNA viruses which include hepatitis B virus^3^. Various stages of the viral life cycle can be targeted by antiviral medications, such as attachment and entry inhibition, uncoating inhibition, replication inhibition, translation inhibition, and release inhibition^4^.

For health care systems around the world, coronavirus disease 2019 (COVID-19) pandemic is a challenge^5^. Drug repurposing, also referred to drug repositioning, involves identifying new therapeutic uses for existing, approved, or investigational drugs that were originally developed for other diseases. This approach has gained substantial attention in recent years due to its ability to significantly shorten the timeline and reduce the cost associated with drug development. Drug repurposing has proven particularly valuable during public health emergencies, such as the COVID-19 pandemic, where rapid therapeutic responses are critical^6–8^. Coronavirus 2 (SARS-CoV-2) is a positive-sense single-stranded RNA virus that first appeared in humans in 2019^9^. SARS-CoV-2 and hepatitis C virus (HCV) share similarities in their replication processes. After the virus is uncoated and enters the cell, it produces nonstructural proteins such as helicase, cysteine, and serine proteases by translating open reading frames 1a and 1b of the positive-strand RNA. Consequently, HCV direct-acting antiviral drugs may be useful against the SARS-CoV-2 virus^10^.

**Fig. 1:**
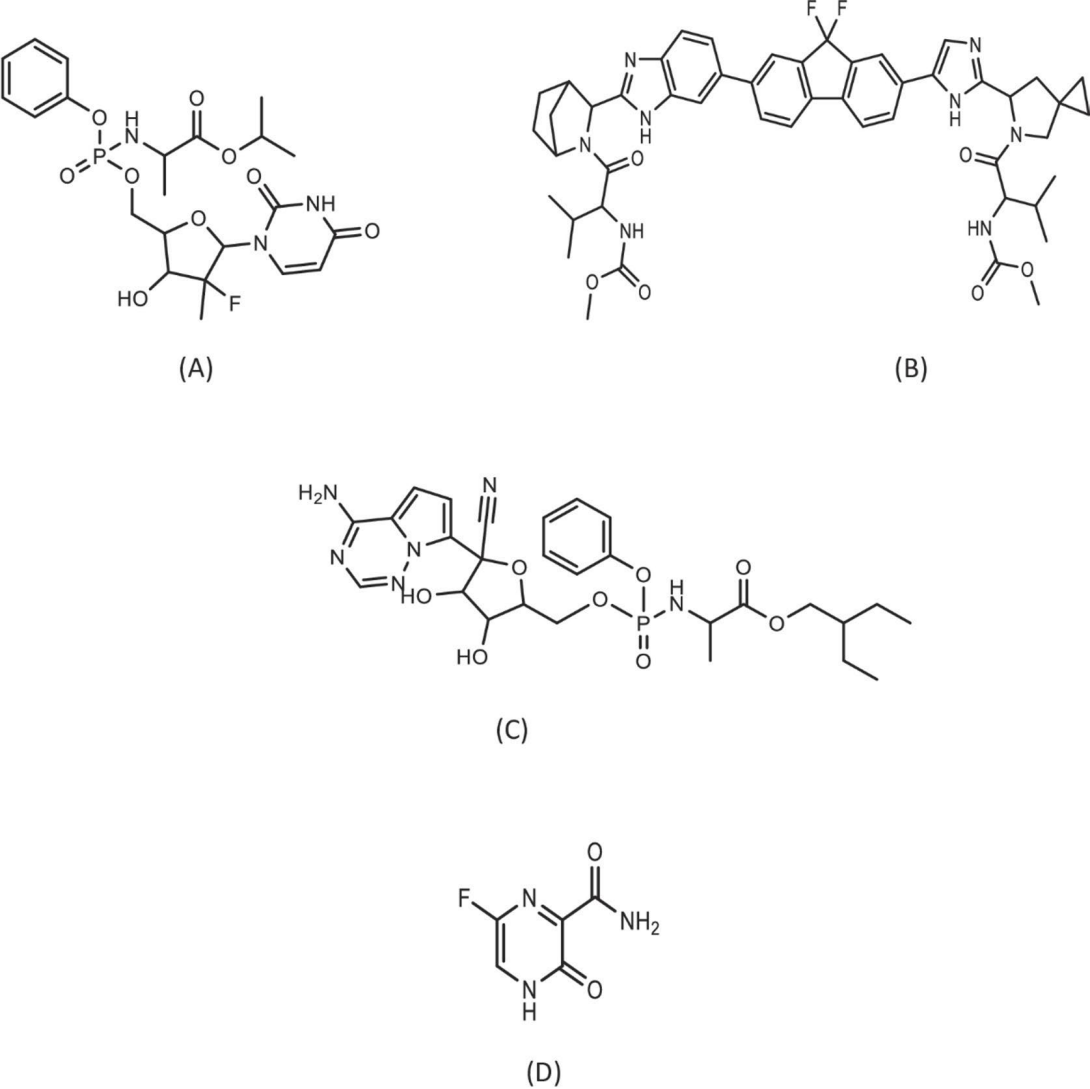
Chemical structures of sofosbuvir (**A**), ledipasvir (**B**), remdesivir (**C**), and favipiravir (**D**).

Sofosbuvir (SOFO, Fig. 1A), an approved direct antiviral medication for HCV, is also effective against other forms of positive-strand RNA viruses. Broad research has been employed on SOFO, a nucleotide analogue inhibitor of the HCV non-structural protein 5B (NS5B) polymerase in individuals infected with genotype-1 HCV who have not received treatment. A significant antiviral efficacy against HCV infections of genotypes 1a and 1b is demonstrated by Ledipasvir (LEDI, Fig. 1B), an inhibitor of NS5A protein. Since this protein plays a role in viral replication, LEDI is expected to limit the viral load in infected patients^11^. Both medications are sold as Ledipasvir 90 mg and Sofosbuvir 400 mg tablets under the trade name Sancovir^®^ 90 mg/400 mg Tablets^12^. As proteins and enzymes necessary for the replication mechanism in SARS-CoV-2 and HCV are nearly identical, combination therapy of SOFO and LEDI may be a viable choice to enhance the treatment of COVID-19 patients. This approach could be especially effective when administered at the early infection and before the virus enter into the parenchymal cells of the lungs^13–16^.

Remdesivir (REMDI, Fig. 1C) is a potent antiviral medication. It is an adenosine analogue monophosphoramidate prodrug. It changes into nucleoside monophosphate (NMP), which undergoes subsequent phosphorylation to active nucleoside triphosphate (NTP) that targets the machinery responsible for of the viral RNA genome’s replication^17–19^. Shortly after its initial synthesis, REMDI was found to possess antiviral action against the Middle East respiratory syndrome coronavirus (MERS-CoV). Therefore, Remdesivir has shown “broad-spectrum” anti-coronavirus activity since the emergence of COVID-19^20,21^.

Favipiravir (FAVI, Fig. 1D) is a prodrug that is metabolically activated in tissues via phosphorylation and ribosylation, resulting in the active metabolite FPV-ribofuranosyl-5-triphosphate. Viral genome RNA transcription and replication are ultimately stopped by FAVI’s method of action, which binds to and inhibits RNA-dependent RNA polymerase (RdRp)^22,23^. Human influenza viruses A, B, and C can be effectively inhibited from reproducing by FAVI, which also works against versions of the virus that are resistant to all anti-influenza medications of practical utility^24,25^. Favipiravir has great potential for COVID-19 patient treatment^26^.

A serious and rapidly expanding risk to the safety of consumers and human wellness is the counterfeiting of pharmaceutical formulations. “A counterfeit drug is one which is purposefully and fraudulently mislabeled with respect to identity and/or source,” according to the World Health Organization’s (WHO) definition, which is the most commonly used. In addition to the risk to public health, pharmaceutical corporations suffer significant financial and brand damages. Counterfeit drugs can also result in untreated medical problems or even death. Numerous measures have been suggested to address the issue of drug counterfeiting. Among these are laws and rules pertaining to illegal sellers, technological countermeasures of drugs either in their pharmaceutical raw form or dosage form, consumer education, and collaboration with authorities^27–29^.

Quality by design (QbD) approach focuses on creating a suitable process and understanding its performance in relation to the desired product outcomes. Traditionally, method development often relies on a trial-and-error approach, where parameters are modified until acceptable results are reached. Nonetheless, this method can be tedious, and resource demanding. QbD tackles these issues by offering a systematic framework for method development^30–32^. In Analytical Quality by Design (AQbD), the development of an analytical method starts with defining the Analytical Target Profile (ATP), which clearly states the objective of the method and the performance standards that must be achieved before conducting any experiments. Following the definition of ATP, Critical Method Attributes (CMAs) are identified as the measurable aspects of method performance such as chromatographic resolution and run time which indicate whether the method meets the defined ATP^33,34^. Two helpful elements of this approach that generate a design framework for the formulation’s input variables are design of experiments (DoEs) and response surface methodology (RSM). A standard experimental design acts by altering one variable at a time; in contrast, the DoE and RSM enable data gathering with fewer runs. In order to evaluate response surfaces, the DoE in conjunction with RSM can fit linear or quadratic functions, producing mathematical representations of output responses as function of input factors^35^. It aids in identifying the reason behind the fluctuating response, establishing the lowest and maximum response conditions, comparing responses across different levels of variables, and creating a response prediction model^36^. One prominent response surface technique that is predictable in identifying the precise interactions of the parameters used in optimization is Box Behnken design (BBD) optimization^37^. BBD is an independent quadratic model where a set of points in the center of each cube surface and duplicate center points define the region of interest^38^. For this investigation, the Box–Behnken design (BBD), which is a three-factor, three-level design, was chosen. BBD also avoids the iso-variance requirement, allowing for the sequential examination of many components. Consequently, BBD is chosen to develop the HPLC analytical method for estimating the mentioned medications^39^.

Various HPLC techniques were developed for the determination of antiviral agents in pharmaceutical formulations. Recent studies have focused on the quantification of FAVI as a single component using conventional or QbD-based approaches^40–42^. Other investigations extended the analysis to binary combinations such as FAVI with REMDI, and SOFO with LEDI^43–45^. Moreover, some studies analyzed multiple antiviral drugs, including one or more of the selected compounds^46–51^. To our knowledge, there isn’t a documented technique for simultaneous determination of the four selected repurposed COVID-19 medications. The objective of the current work is to develop and validate a consistent, accurate method for the concurrent measurement of FAVI, SOFO, REMDI, and LEDI which will be utilized for their determination in their respective dosage forms. It enables the simultaneous separation and quantification of the four pharmaceutical compounds in a single analytical run, which significantly reduces the time of analysis, and solvent consumption compared to other reported methods. Also, applying a QbD approach ensures a systematic understanding of the method’s variables and their impact on chromatographic separation, leading to a robust and reliable analytical procedure. Further assessment of the method’s sustainability was achieved via greenness, blueness, and whiteness evaluation using AGREE, MoGAPI, BAGI, and RAPI tools.

## Experimental

### Instrument

The experimental setup employed an Agilent 1100 series chromatographic system. The configuration consisted of a G1310A isocratic pump, G1314A UV-visible detector, and G1328B manual injector featuring a (20 µl) injection loop, all from Agilent. Analysis was performed using an INERTSIL ODS-3 C18 column (5 μm, 250 × 4.6 mm). The setup included a sonicator (Power Sonic 405, Human Lab, Gwangju-si, Gyeonggi, Korea) and pH meter (Jenway, 3505, Essex, UK). The mobile phase underwent filtration using nylon membrane filters (0.45 μm pore size) from Sigma-Aldrich Co. in Germany.

### Materials and reagents

FAVI and REMDI were kindly supplied by Eva Pharma (Egypt), while SOFO and LEDI were gifted by Marcyrl Pharmaceutical Industries (Egypt). The certified purities were 99.76, 100.47, 100.13, and 100.43% for FAVI, SOFO, REMDI, and LEDI, respectively. HPLC grade acetonitrile and ammonium formate were acquired from Sigma-Aldrich, Darmstadt, Germany. Bi-distilled water was obtained in-house using Aquatron Water Still (A4000D, Staffordshire, UK). Sancovir^®^ tablets (batch no. 214022), containing 200 mg of FAVI per tablet, Remdesivir EVA^®^ vials (batch no. 212948), containing 5 mg/mL of REMDI per vial, Sofolanork Plus^®^ tablets (batch no. M1047918), containing 400 mg of SOFO and 90 mg of LEDI per tablet were procured from the market.

### Stock solutions

Precise quantities of FAVI, SOFO, REMDI and LEDI was individually placed into a 100 mL volumetric flask, which was subsequently filled to mark with acetonitrile for FAVI, SOFO, and REMDI and with mobile phase for LEDI to prepare standard stock solutions (1 mg/mL).

### Experimental design for optimization of chromatographic conditions

A three-level Box–Behnken design incorporating a center point was employed to enhance the key parameters influencing HPLC separation. The experimental protocol comprising fifteen runs, conducted by introducing a mixture of the specified medications, is detailed in Table 1. The selected responses—resolution between initial drug pair (R1), resolution between middle drug pair (R2), resolution between final drug pair (R3), and runtime (RT)—were documented and utilized for model development. Each trial represented a distinct combination of factor levels. The investigated factors included acetonitrile ratio, buffer pH, and flow rate, each examined at three levels. Data evaluation was conducted using Minitab^®^ 17 statistical software (Minitab, Inc., State College, Pennsylvania, USA). The resulting model was expressed through a mathematical second-order equation encompassing individual, interactive, and quadratic components. The ideal chromatographic parameters for optimal response were determined using response surface and contour plots. Model validation involved residual plots, lack-of-fit testing, and analysis of variance (ANOVA).

**Table 1:** Experimental matrix and experimental plan of the Box–Behnken design.

No. of experiments	Experimental variables			Buffer pH	Organic ratio (%)	Flow rate
	X_1_	X_2_	X_3_			
1	1	− 1	0	4	40	1
2	1	0	− 1	4	50	0.8
3	− 1	− 1	0	2.5	40	1
4	− 1	1	0	2.5	60	1
5	0	0	0	3.25	50	1
6	0	− 1	− 1	3.25	40	0.8
7	0	1	1	3.25	60	1.2
8	0	− 1	1	3.25	40	1.2
9	0	0	0	3.25	50	1
10	0	1	− 1	3.25	60	0.8
11	− 1	0	− 1	2.5	50	0.8
12	− 1	0	1	2.5	50	1.2
13	1	0	1	4	50	1.2
14	1	1	0	4	60	1
15	0	0	0	3.25	50	1

### Developed chromatographic conditions

An INERTSIL ODS-3 C18 column (5 mm, 250 × 4.6 mm) was utilized as the stationary phase for the LC separation and quantification. The mobile phase was comprised of acetonitrile (50:50, v/v) and ammonium formate buffer (0.02 M, adjusted to pH 2.8 with formic acid) at a flow rate of 1 mL/min. After passing through a 0.45 μm membrane filter, the mobile phase was degassed in an ultrasonic bath. Before the solutions were injected, the system was allowed to settle and become saturated with the mobile phase for 30 min. The analysis was done at room temperature. Peak area using UV detection at λ 240 nm was used for quantification.

### Laboratory prepared mixtures

To prepare different combinations with varying proportions of FAVI, SOFO, REMDI, and LEDI, precise portions of the working solutions were pipetted into multiple 10 mL volumetric flasks, brought to volume with acetonitrile, and thoroughly homogenized.

### Sample preparation

Twenty tablets of Sancovir^®^ and Sofolanork Plus^®^ were individually weighed and coarsely ground. A measured amount of the ground Sancovir^®^ tablets containing 50 mg of FAVI, also a measured amount of the ground Sofolanork Plus^®^ containing 50 mg of SOFO, and 11.25 mg of LEDI was accurately weighed. Subsequently 30 mL of (ACN: H_2_O) mixture in 50:50 ratio was added for FAVI, while the established mobile phase was used for SOFO and LEDI. Each solution was subjected to 15 min of sonication for drug extraction. The volume was brought to 50 mL using the same extraction solvent to prepare a stock solution (1 mg/mL) of FAVI, SOFO, and LEDI individually. Each solution underwent thorough mixing and filtration through a dry funnel and filter paper, with the initial few milliliters being discarded. Further dilution using acetonitrile was performed to prepare sample working solutions of varying concentrations. For REMDI working solution preparation (100 mg/mL), 1 mL of remdesivir vial content was diluted with methanol in a 50 mL volumetric flask. Additional dilution using the established mobile phase was done to create sample working solutions of different concentrations.

### Validation of the proposed method

The established RP-HPLC method was tested for linearity, accuracy, precision, limit of detection (LOD), limit of quantitation (LOQ), and system suitability parameters in accordance with ICH guideline Q2 (R2) for simultaneous determination of FAVI, SOFO, REMDI, and LEDI in pharmaceutical products.

## Results and discussion

### Optimization of chromatographic conditions using BBD design

The ATP of the present study was to develop a chromatographic method capable of providing adequate separation of the four analytes, ensuring a resolution not less than 2 and acceptable run time suitable for routine analysis, while maintaining satisfactory quantitative performance with accuracy within 98–102% and precision (RSD ≤ 2%). Based on the ATP, resolution and run time were identified as CMAs, as they directly reflect the separation efficiency and analysis time of the method. Accordingly, the experimental BBD was utilized to optimize the conditions of the HPLC technique. Since BBD makes it possible to estimate the quadratic model’s parameters, create sequential designs, and identify the model’s lack of fit, it is a valuable tool in RSM. BBD offers appropriate mathematical models that require fewer experimental runs. The developed models demonstrate how each factor influences the experiment’s result and determines the optimal values for each factor. The factors assessed were acetonitrile ratio, flow rate, and pH of the buffer, while selected responses were resolution between initial drug pair (R1), resolution between middle drug pair (R2), resolution between final drug pair (R3), and runtime (RT). Fifteen trials were performed utilizing the levels shown in Table 1. The experimental data were fitted to a second-order polynomial equations, and the coefficients of the response model were found. These trials were designed following BBD matrix^52–57^. The correlation between the variables and the expected response is expressed using linear, quadratic, and interaction terms as follows:

Regression equation for R1 (Eq. 1):

1 $$\begin{aligned} {\mathrm{R}}1 & =57.66-1.946\% {\mathrm{ACN}}+7.07\;{\mathrm{flow}}\;{\mathrm{rate}}+0.01694\% {\mathrm{ACN}}*\% {\mathrm{ACN}} \\ & \quad - 0.1262\% {\mathrm{ACN}}*{\mathrm{flow}}\;{\mathrm{rate}} \\ \end{aligned}$$

Regression equation for R2 (Eq. 2):

2 $$\begin{aligned} {\mathrm{R}}2 & = - 21.80+40.73\;{\mathrm{buffer}}\;{\mathrm{pH}} - 1.396\% {\mathrm{ACN}} - 4.355\;{\mathrm{buffer}}\;{\mathrm{pH}}*{\mathrm{buffer}}\;{\mathrm{pH}} \\ & \quad +0.01613\% {\mathrm{ACN}}*\% {\mathrm{ACN}} - 0.1717\;{\mathrm{buffer}}\;{\mathrm{pH}}*\% {\mathrm{ACN}} \\ \end{aligned}$$

Regression equation for R3 (Eq. 3):

3 $$\begin{aligned} {\mathrm{R}}3 & = - 52.9+32.39\;{\mathrm{buffer}}\;{\mathrm{pH}}+0.256\% {\mathrm{ACN}} - 3.089\;{\mathrm{buffer}}\;{\mathrm{pH}}*{\mathrm{buffer}}\;{\mathrm{pH}} \\ & \quad - 0.1487\;{\mathrm{buffer}}\;{\mathrm{pH}}*\% {\mathrm{ACN}} \\ \end{aligned}$$

Regression equation for RT (Eq. 4):

4 $$\begin{aligned} {\mathrm{RT}} & = 146+122.7\;{\mathrm{buffer}}\;{\mathrm{pH}} - 11.87\% {\mathrm{ACN}}+0.1616\% {\mathrm{ACN}}*\% {\mathrm{ACN}} \\ & \quad - 2.080\;{\mathrm{buffer}}\;{\mathrm{pH}}*\% {\mathrm{ACN}} \\ \end{aligned}$$

where R₁, R₂, and R₃ represent the resolution between the first, second, and third pairs of analyte peaks, respectively. RT refers to the total chromatographic run time. %ACN is the percentage of acetonitrile in the mobile phase, representing the organic modifier. pH refers to the pH of the aqueous buffer component of the mobile phase. The terms such as %ACN × %ACN and pH × pH indicate quadratic effects, reflecting nonlinear relationships, while interaction terms like pH × %ACN represent interaction effects between the two factors. Coefficients reflect the magnitude and direction of these effects: positive coefficients indicate direct proportionality, whereas negative coefficients suggest inverse relationships. This model incorporates linear effects, quadratic effects, and the interactions among the factors being studied.

### Effect of factors

Equation (1) shows that R1, the resolution of FAVI from SOFO, increases with flow rate but decreases with higher ACN ratio. The positive quadratic effect (+ 0.01694) means the impact of %ACN on resolution is not strictly linear. After a certain point, further increases in %ACN may reduce the negative impact or even improve resolution, suggesting that there is an optimal %ACN for the optimum peak separation. The negative interaction term (− 0.1262) implies that the simultaneous increase of both %ACN and flow rate has a smaller effect on resolution than increasing each one alone. So, while each factor individually enhances or diminishes resolution, their combined effect is antagonistic within the studied region.

Equation (2) reveals that R2, the resolution of SOFO from REMDI, increases with buffer pH but decreases with higher ACN ratio. The large positive linear coefficient (+ 40.73) suggests that increasing buffer pH initially increases the resolution between the second and third peaks. However, the significant negative quadratic term (− 4.355) of buffer pH implies a parabolic relationship; after a certain optimum pH, further increases in pH reduce resolution. This behavior reflects the pH sensitivity of the analytes and suggests the existence of an optimal pH range for maximal resolution. The positive quadratic term of ACN ratio (+ 0.01613) imply that although resolution decreases at moderate %ACN levels, very high or low %ACN values may partially restore resolution. The negative interaction term shows an antagonistic effect for the combined factors.

The positive linear coefficient (+ 32.39) of buffer pH in Eq. (3) indicates that increasing buffer pH improves resolution between peaks of REMDI and LEDI. The negative quadratic term (− 3.089) suggests that there is an optimal pH beyond which resolution declines. The tiny positive coefficient for %ACN (+ 0.256) suggests a slight improving effect on R3 with increasing acetonitrile. The negative interaction coefficient (− 0.1487) indicates a negative interaction between pH and %ACN. This means that the beneficial effect of increasing one factor (e.g., pH) is partially offset when the other factor (%ACN) is also increased, and vice versa. This illustrates a less-than-additive effect in which an increase in both variables at the same time reduces the resolution gain.

The strong positive linear coefficient (+ 122.7) in Eq. (4) suggests that increasing the buffer pH causes a significant increase in run time. The negative linear coefficient (− 11.87) of ACN ratio indicates that increasing %ACN significantly reduces run time, which aligns with the expected behavior in reversed-phase chromatography, where higher organic content enhances elution strength and shortens retention. The positive quadratic coefficient (+ 0.1616) for the ACN ratio indicates that the run time rises as the factor level diminishes until reaching a critical threshold, beyond which additional decreases result in an increase in the response. The negative interaction term (− 2.080) suggests a negative interaction between the combined factors.

**Fig. 2:**
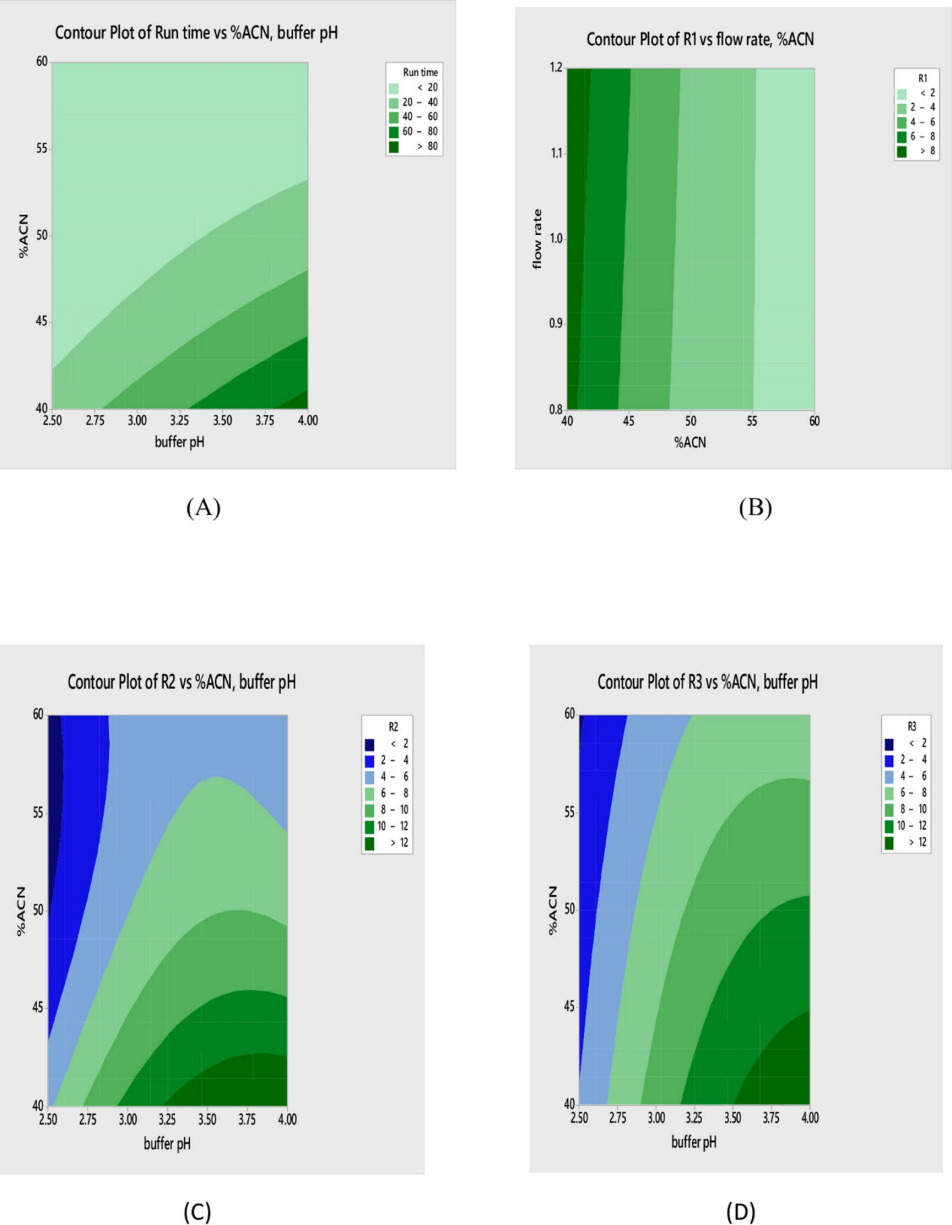
Contour plots showing the effect of the selected factors on Run time, R1, R2, and R3.

**Fig. 3:**
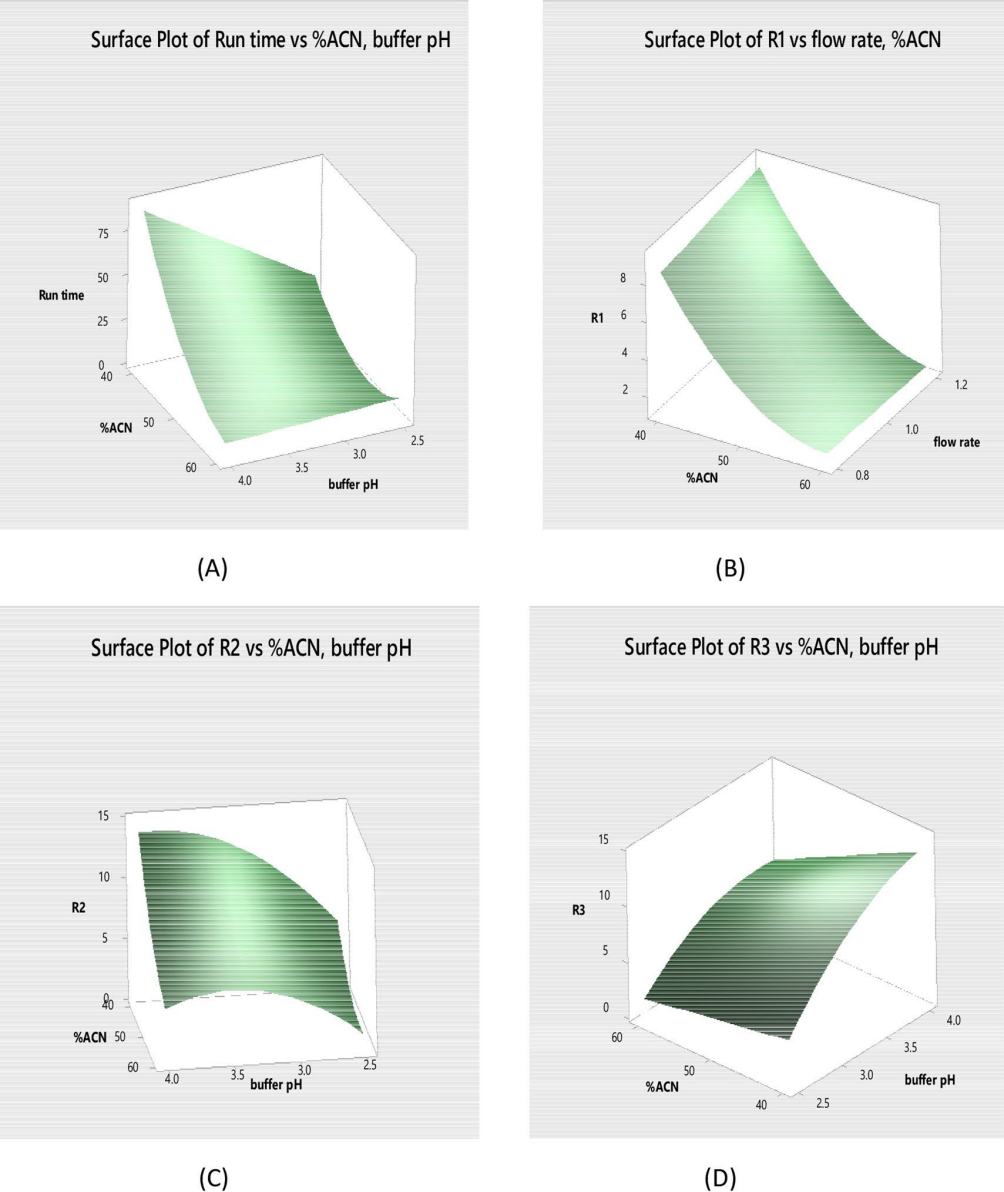
Response surface plots showing the effect of the selected factors on Run time, R1, R2, and R3.

The quadratic polynomial equations which were generated using Box–Behnken design to capture the relationships between chromatographic factors and key responses are illustrated graphically in Figs. 2 and 3 via two-dimensional contour maps and three-dimensional response surface graphs, respectively. The model integrated linear terms reflecting individual factor effects, squared terms accounting for curvature, and interaction terms to describe combined factor influences. The shapes of these plots revealed curvature indicative of non-linear responses and inter-factor interactions. Moreover, the highest response values can be visually located at the apex of the surface or the center of contour patterns guiding precise identification of optimal analytical conditions^58–60^.

### Desirability-based numerical optimization for optimum separation conditions

**Fig. 4:**
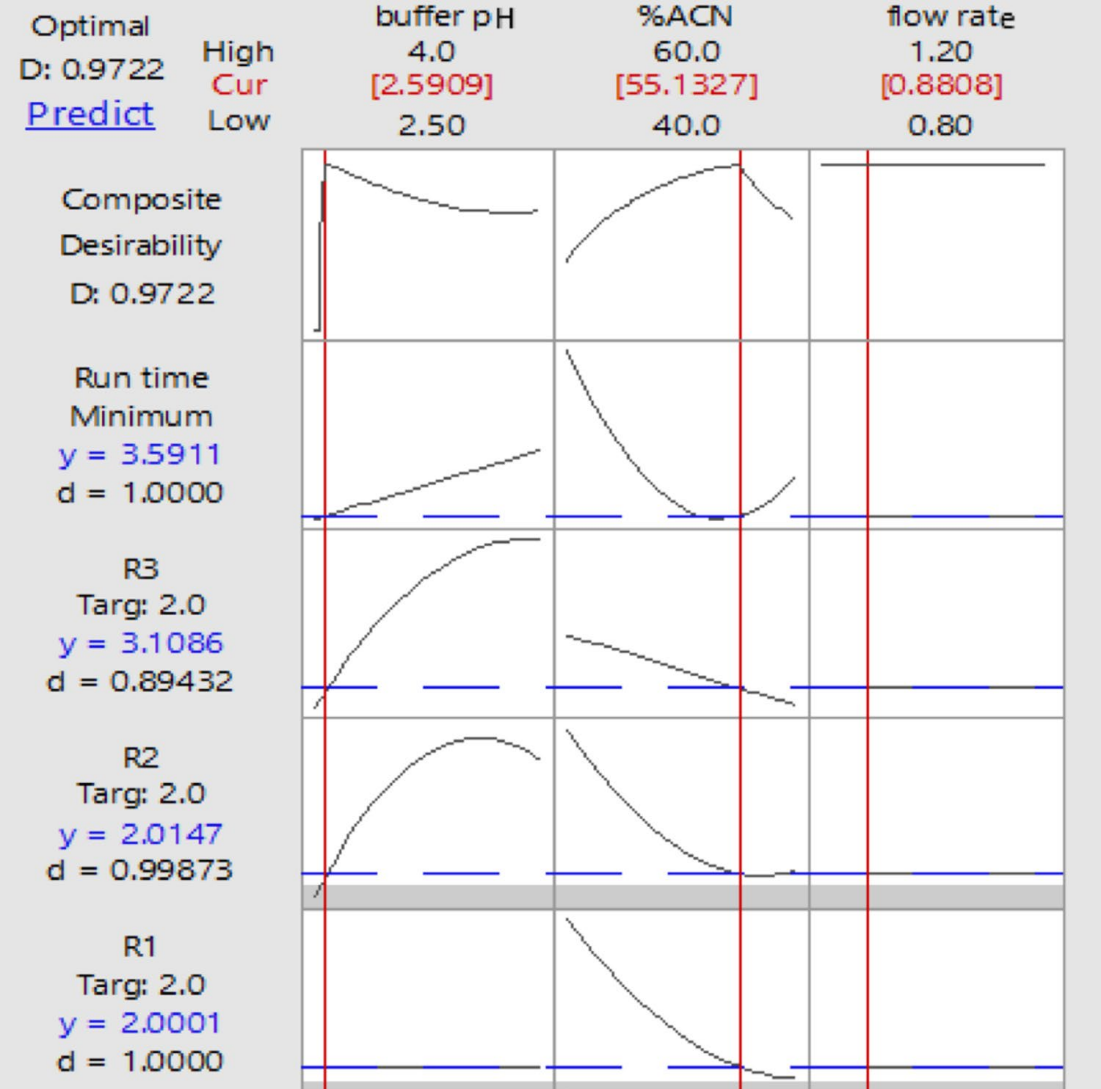
Response optimization showing the calculated desirability factor of the used responses.

Based on the response optimizer tool and the final fitted models, criteria were defined to optimize each response and select the best experimental conditions for analyzing the laboratory-prepared mixture. The optimal conditions were those that provided the most favorable combined values of R1, R2, R3, and RT. The goal was to achieve the best peak resolution by targeting R1 = R2 = R3 = 2 while minimizing RT. The response optimizer determined the optimum settings and produced the optimization plot, Fig. 4.

### Statistical analysis of the models

The experimental data was statistically analysed using Minitab^®^ 17 statistical software (Minitab, Inc., State College, Pennsylvania, USA)^61^. The quality of the model was evaluated using ANOVA (analysis of variance)^62^. From the results of ANOVA (Table 2), the overall regression models were statistically significant (*p* < 0.05), confirming the adequacy of the fitted quadratic models for describing the relationship between the studied factors and the responses. Examination of the individual model terms revealed that pH and organic modifier composition (%ACN) exerted statistically significant effects on most of the measured responses (*p* < 0.05). In contrast, the linear effect of flow rate showed a p-value of 0.060, indicating that within the investigated experimental range, its influence was not statistically significant. Several interaction and quadratic terms, including %ACN × flow rate, pH², and pH × %ACN, were statistically significant for specific responses, highlighting the presence of curvature and interaction effects within the studied design space^63^. The coefficient of determination (R^2^) quantifies the proportion of variance explained by the model. It is a statistical indicator of the degree of fit and it turned out to fall within acceptable limits (R^2^ ≥ 80%)^64^. A high adjusted R-square value indicates a significant correlation between experimental data and the fitted models. As presented in (Table 3), for every response, there was an adequate agreement between the adjusted R-square value and the predicted R-square value.

**Table 2:** Analysis of variance (ANOVA) results of the model.

Source of variation	Run time *p*-Value	R1 *p*-Value	R2 *p*-Value	R3 *p*-Value
Model	0.000	0.000	0.000	0.000
Constant	0.000	0.000	0.000	0.000
pH	0.000	–	0.000	0.000
Flow rate	–	0.060	–	–
Organic ratio	0.000	0.000	0.000	0.000
%ACN*%ACN	0.000	0.000	0.000	–
buffer pH*buffer pH	–	–	0.000	0.004
%ACN*flow rate	–	0.032	–	–
buffer pH*%ACN	0.000	–	0.000	0.035
Lack-of-fit	0.006	0.241	0.045	0.003

**Table 3:** Models fitting results.

Model term	Run time	R1	R2	R3
R^2^	95.75	99.67	99.38	95.16
Adjusted R^2^	94.06	99.54	99.03	93.22
Predicted R^2^	81.77	99.08	96.87	79.86

Model adequacy was assessed using the lack-of-fit test^65^. While R2 showed non-significant lack-of-fit (*p* > 0.05), significant lack-of-fit was observed for Run time (*p* = 0.006) and R3 (*p* = 0.003), indicating that the quadratic model may not fully capture variability in these responses. This may be due to experimental noise or higher-order effects not included in the design. While the model provided practical reliability at the optimum point, its overall predictive capacity was affected by the inherent chromatographic challenges of the separation. However, the overall models were statistically significant, residual analysis showed no systematic trends, and experimental validation confirmed good agreement between predicted and observed values, supporting the practical reliability of the models.

### Residual analysis

**Fig. 5:**
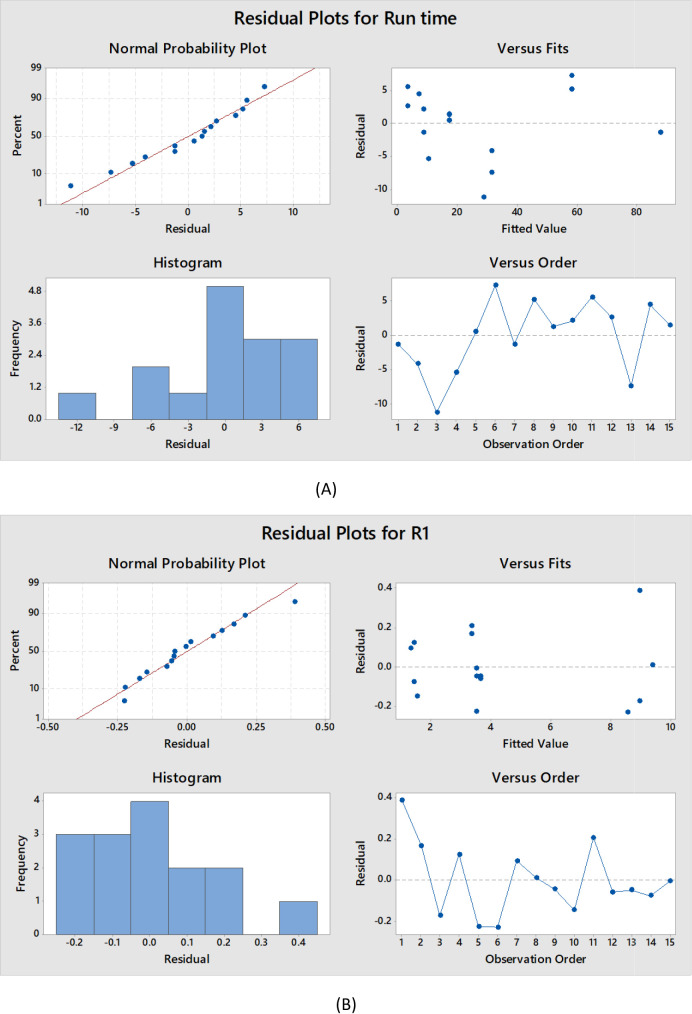

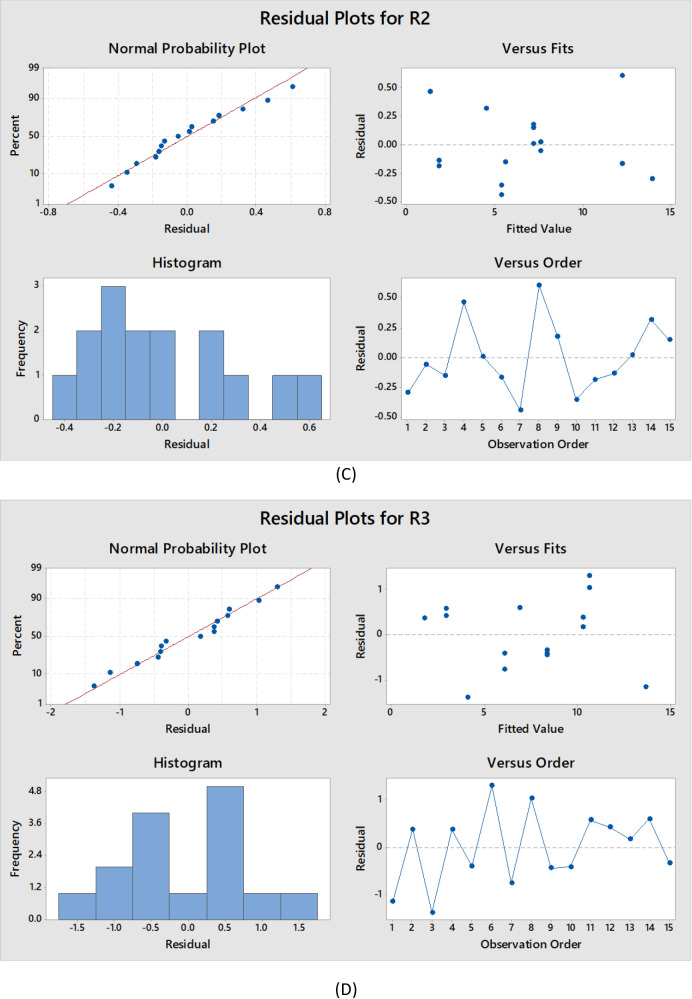
Normal probability plot, histogram, versus fits and versus order for Run time, R1, R2, and R3.

Residual analysis was performed to assess the adequacy of the polynomial models generated via Box–Behnken design. As illustrated in Fig. 5, the normal probability plot exhibited points that aligned approximately along a straight line, indicating that the residuals followed a normal distribution pattern. The histogram of residuals appeared symmetric and oriented around zero, which supports the normality assumption. The plot of residuals against fitted values demonstrated a random distribution of points on both sides of the zero-line, constant variance through the predicted values’ range. Furthermore, the residuals versus run order plot showed that the residuals were randomly dispersed, implying independence of errors across experiments. These graphical diagnostics collectively confirm that the regression assumptions were reasonably met^66–69^.

### Determination of the optimal separation conditions

**Fig. 6:**
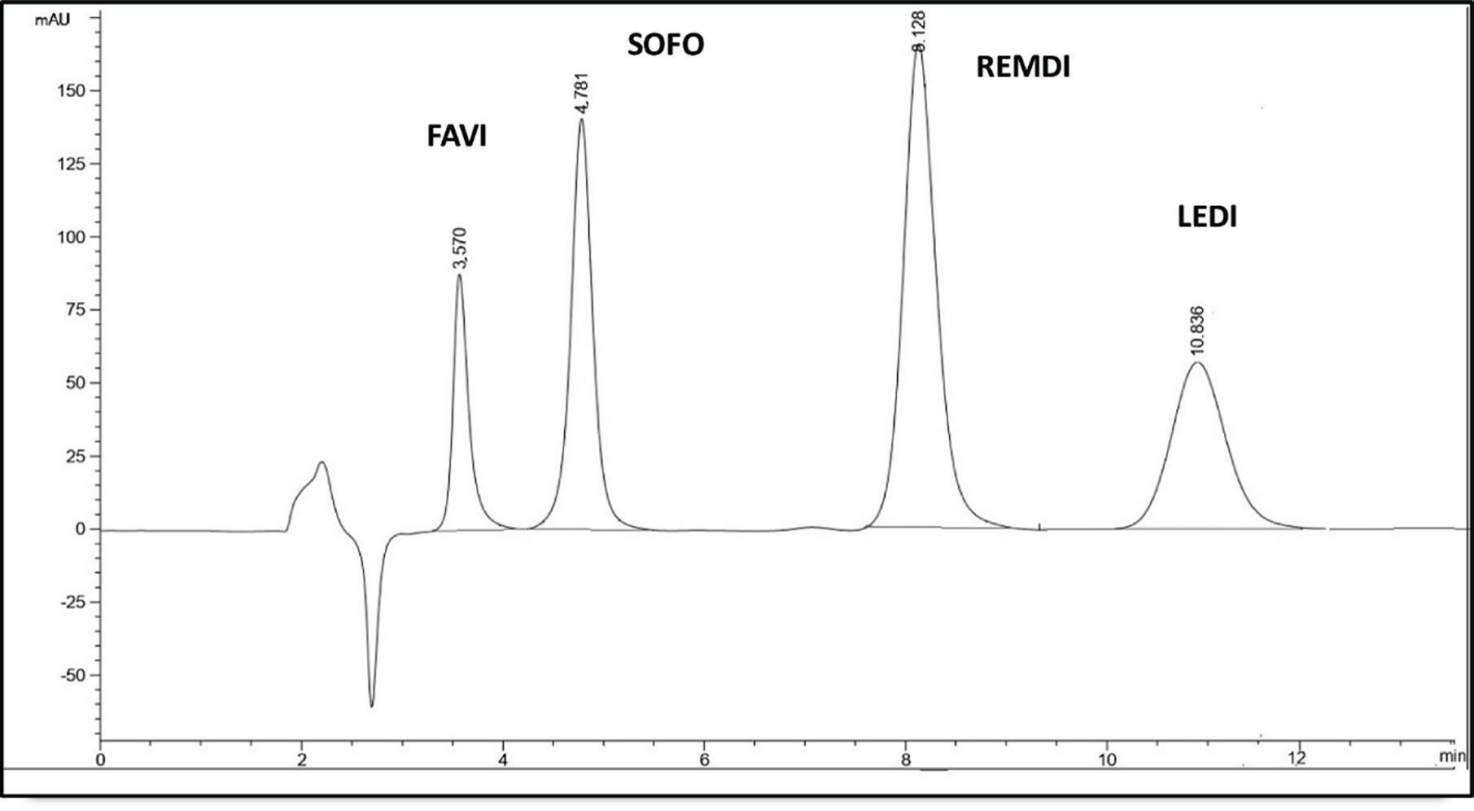
HPLC chromatogram of a laboratory prepared mixture of FAVI, REMDI, LEDI and Sofo using the developed method.

The purpose of adjusting the chromatographic separation was to identify the settings that yielded the optimal values of R1, R2, R3, and run time (RT) owing to the final models generated^70^. The quadratic models and interaction effects were utilized in order to analyze the data^71^. Table 2 displays the optimized model parameters. The mobile phase composition of the optimized solution included acetonitrile and 0.02 M ammonium formate buffer (pH 2.8), (50:50, v/v) and the flow was 1 ml/min using an isocratic mode. Symmetric peaks and adequate separation were obtained using these conditions. The chromatogram is displayed in Fig. 6. With approximate retention times of 3.750, 4.781, 8.128, and 10.836, respectively, the investigated compounds were eluted in the following order: FAVI, SOFO, REMDI, and LEDI.

### Method validation

#### Linearity

Linearity was assessed by constructing calibration curves for FAVI, SOFO, REMDI, and LEDI at six concentration levels, covering the studied concentration range. The peak areas were graphed as a function of their respective concentrations, and the regression analysis yielded correlation coefficients 0.9999, 0.9999, 0.9997, 0.9999 for FAVI, SOFO, REMDI, and LEDI, respectively. Linear relationship was achieved over the concentration range (2–100 µg/mL) for FAVI, REMDI, and LEDI, and (8–400 µg/mL) for SOFO. Table 4 shows the outcomes derived from the calibration curves including standard deviations for the slope (Sb) and intercept (Sa).

**Table 4:** Validation parameters and results obtained by the developed RP-HPLC method.

Item	FAVI	SOFO	REMDI	LEDI
Retention time (tR) (min)	3.570 min	4.781 min	8.128 min	10.836 min
Wavelength of detection (nm)	240 nm	240 nm	240 nm	240 nm
Range of linearity	2–100 µg/mL	8–400 µg/mL	2–100 µg/mL	2–100 µg/mL
Regression equation	AUP 240 nm	AUP 240 nm	AUP 240 nm	AUP 240 nm
	= 20.483 C_FAVI_	= 10.519 C_SOFO_	= 76.447 C_REMDI_	= 48.834 C_LEDI_
	+ 4.9577	+ 22.174	− 0.6995	− 1.7364
Regression coefficient (r^2^)	0.9999	0.9999	0.9997	0.9999
LOD (µg/mL)	0.348	0.570	0.092	0.102
LOQ (µg/mL)	1.036	1.728	0.278	0.310
Standard deviation of slope (Sb)	0.107525	0.056702	0.613044	0.175438
Standard deviation of the intercept (Sa)	5.532051	11.66895	31.54048	9.026122
Confidence limit of the slope	20.2071±20.7599	10.3732 ± 10.6647	74.8711 ± 78.0229	43.3832 ± 44.2852
Confidence limit of the intercept	− 9.6229 ± 18.8183	− 7.8224 ± 52.1695	− 81.7769 ± 80.3778	− 24.9388 ± 21.4659
Standard error of estimate	10.2535	21.6281	58.4595	16.7297
Intra-day RSD (%)	0.088–0.250	0.059–0.181	0.019–0.063	0.046–0.402
Inter-day RSD (%)	0.081–0.503	0.014–0.058	0.011–0.189	0.025–0.222
Drug in bulk	100.015 ± 0.857	100.112 ± 0.762	99.281 ± 0.963	99.241 ± 0.895
Drug in dosage form	100.540 ± 0.402	100.745 ± 0.444	100.709 ± 0.707	100.628 ± 0.592
Drug added	99.804 ± 0.526	100.423 ± 0.539	100.058 ± 0.300	100.300 ± 0.383

^a^Limits of detection and quantification are determined via calculations: LOD = 3.3 × SD/slope, LOQ = 10 × SD/slope.

^b^The intraday (*n* = 3), average of three concentrations of FAVI, REMDI, LEDI (12, 50 and 75 µg/mL) and of SOFO (48, 200 and 300 µg/mL) repeated three times within the day.

^c^The interday (*n* = 3), average of three concentrations of FAVI, REMDI, LEDI (12, 50 and 75 µg/mL) and of SOFO (48, 200 and 300 µg/mL) repeated three times in three successive days.

#### Accuracy

The accuracy of an analytical method is defined as its capacity to yield test results that are closely approximate to the true value. In this study, accuracy was assessed by calculating recoveries at 6 distinct concentration levels. Each level was analyzed in triplicate. As shown in Table 4, recoveries of the drugs exceeded 98%.

#### Limit of detection and limit of quantitation

In accordance with ICH guidelines^72^, both limit of detection (LOD) and limit of quantitation (LOQ) were estimated using the regression equation’s slope and the response’s standard deviation and results are presented in Table 4.

#### Precision

Precision was established by assessing repeatability (intra-day) and intermediate precision (inter-day). Intra-day precision was determined by analyzing six replicates at three concentration levels within a single day, while inter-day precision was evaluated over three consecutive days. As stated in Table 4, the calculated %RSD values were consistently below 2% for all analytes, confirming that the method can produce consistent results under normal operating conditions.

#### System suitability tests

The system suitability features were described using plate number, resolution factor, tailing factor, capacity factor, and selectivity factor. It was confirmed by making six consecutive injections of 20 µg/mL solution. Table 5 reports the test results for the developed approach.

**Table 5:** System suitability tests for the developed RP-HPLC method.

Parameter	FAVI	SOFO	REMDI	LEDI	Reference value
N	3039	2567	3166	1613	The higher the value, the more efficient the column is
R		3.86	7.17	3.11	> 2
T	0.70	0.95	0.82	0.92	≤ 2
K’	0.428	0.912	2.251	3.334	1–10
*α*		1.35	1.72	1.32	≥ 1

*N* number of theoretical plates, *R* resolution, *K*′ capacity factor, *α* selectivity factor, *T* tailing factor.

#### Specificity

**Fig. 7:**
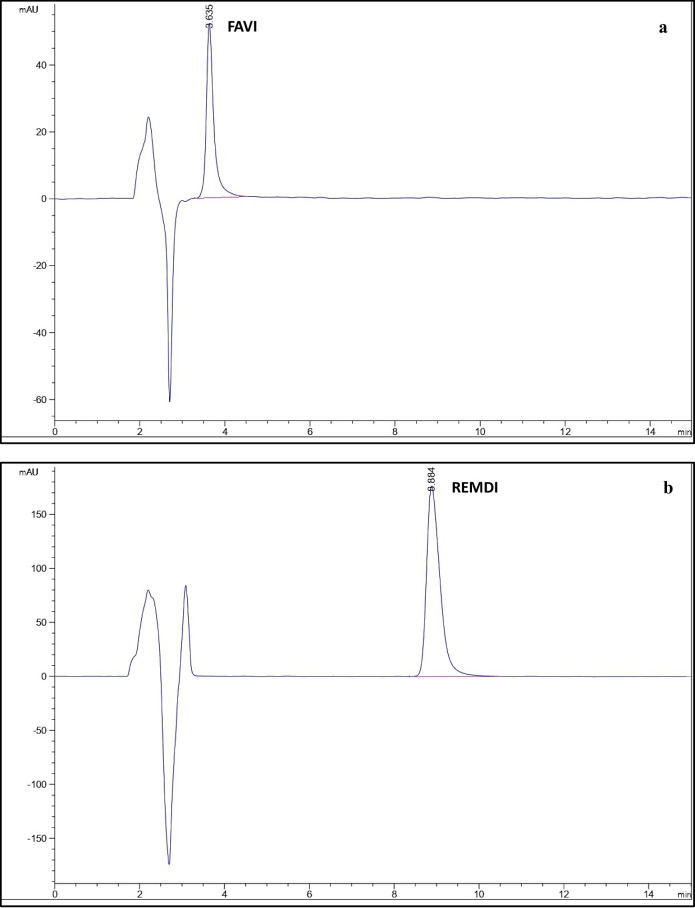

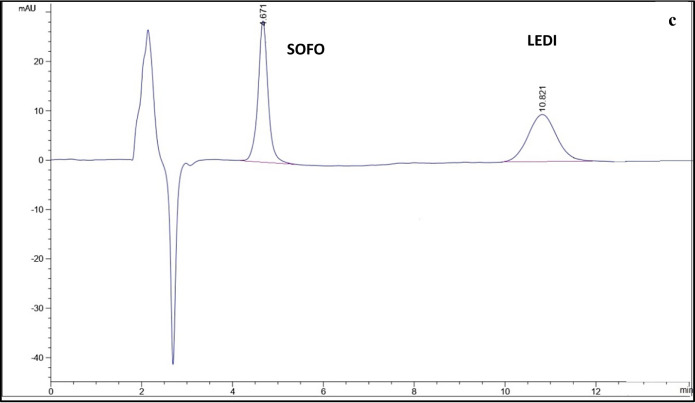
HPLC chromatograms using the developed method of (**a**) FAVI in Sancovir^®^ tablet, (**b**) REMDI in Remdesivi EVA^®^ vial, (**c**) SOFO and LEDI in Sofolanork Plus^®^ tablet.

The capacity of an analytical method to precisely measure the analyte in the presence of potentially anticipated components in the sample matrix is known as specificity. It was assessed to make sure that the excipients in the formulations weren’t interfering. By determining each medication independently in its dosage form, specificity was investigated; no excipient influence was found, as shown in Fig. 7. Moreover, in Table 6, specificity was achieved by analyzing the stated medications in laboratory-prepared mixes with various proportions of the listed drugs.

**Table 6:** Determination of FAVI, SOFO, REMDI, and LEDI in laboratory prepared mixtures using the proposed RP-HPLC method.

Taken (µg/ml)				AUP				Found (µg/ml)				Recovery %			
FAVI	REMDI	LEDI	SOFO	FAVI	REMDI	LEDI	SOFO	FAVI	REMDI	LEDI	SOFO	FAVI	REMDI	LEDI	SOFO
8	8	8	32	168.175	607.742	347.002	356.821	7.986	7.959	7.956	31.814	99.825	99.487	99.449	99.417
17	17	17	68	348.416	1279.377	749.536	733.248	16.786	16.745	17.139	67.777	98.738	98.498	100.818	99.673
25	25	25	100	513.049	1886.221	1088.048	1066.436	24.823	24.683	24.537	99.274	99.292	98.731	98.146	99.274
45	45	45	180	936.417	3376.023	1965.494	1916.435	45.492	44.171	44.879	180.080	101.094	98.157	99.731	100.044
85	85	85	340	1747.350	6564.997	3683.483	3643.032	85.083	85.886	84.072	344.221	100.097	101.042	98.908	101.241
95	95	95	380	1970.735	7245.312	4095.480	4060.164	95.989	94.785	93.471	383.876	101.041	99.773	98.391	101.020
Mean												100.015	99.281	99.241	100.112
SD												0.857	0.963	0.895	0.762
SE												0.350	0.393	0.365	0.311

#### Integrated sustainability evaluation of the proposed method

Assessing the sustainability of analytical methods has become crucial to comprehend their efficiency, safety, environmental friendliness, and financial affordability. While Green and White Analytical Chemistry address environmental friendliness and analytical performance, respectively, the inclusion of Blue Analytical Chemistry emphasizes the applicability of the developed methods^73–75^. Our proposed method was evaluated using established assessment tools, including AGREE, and MoGAPI for its greenness, BAGI for its blueness, and RAPI for its whiteness.

### Greenness assessment

#### Analytical greenness tool (AGREE)

**Fig. 8:**
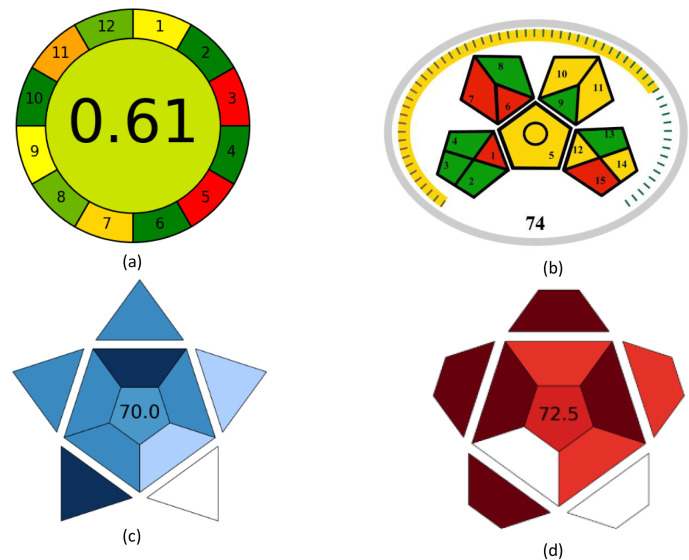
Sustainability evaluation using: (**a**) AGREE, (**b**) MoGAPI, (**c**) BAGI, (**d**) RAPI.

AGREE is a simple tool introduced in 2020, for evaluating the environmental impact of analytical methods. The assessment is represented as a pictogram with twelve sectors, each represents a concept of the Green Analytical Chemistry (GAC). Each sector is graded on a scale of 0 to 1. One represents entirely green and zero represents non-green, and the color of each sector can shift from dark green to red with an overall score in the center of the pictogram that reflects the greenness of the method^76–78^. As shown in Fig. 8a, our proposed method demonstrated an AGREE score of 0.61. It should be noted that some reported methods may exhibit shorter run times or higher AGREE score, however these methods typically analyze one or two drugs, while the proposed method simultaneously determine the four drugs.

#### Modified green analytical procedure index (MoGAPI)

MoGAPI has been developed in 2024 as an enhanced tool for greenness evaluation, combining the visual color-coded pictogram of the original GAPI with an overall greenness score. The MoGAPI pictogram consists of interconnected pentagons, each reflect the environmental impact of specific analytical parameters, where green represents low impact, yellow denotes moderate impact, and red indicates high environmental impact^79–82^. Figure 8b, illustrates the MoGAPI pictogram of the proposed method, in which red fields correspond to offline operation, macroextraction procedures, the use of acetonitrile in the mobile phase, and the absence of waste treatment step. The predominance of green and yellow fields with a greenness score of 74 indicates the favorable environmental profile.

### Blueness assessment

#### Blue applicability grade index (BAGI)

BAGI is a recently introduced tool designed to evaluate the practical applicability of analytical methods based on ten criteria covering the nature of the analysis, multi-analyte capability, instrumentation, sample handling capacity, sample preparation, the number of samples analyzed per hour, reagents and materials, preconcentration requirements, automation level, and sample volume. The BAGI output is presented as a schematic diagram composed of graded fields, where darker blue regions indicate higher applicability accompanied by a numerical score^83–85^. As illustrated in Fig. 8c, the BAGI evaluation resulted in an overall score of 70 indicating good method applicability.

### Whiteness assessment

#### Red analytical performance index (RAPI)

RAPI has been developed to provide a comprehensive assessment of the overall quality of analytical methods based on key validation parameters. Each validation parameter contributes to a numeric score ranging from 0 to 10. The RAPI pictogram allows a quick visual assessment of analytical performance, where the color varies gradually from white for low performance to darker shades of red for higher performance with an overall score from 0 to 100^86–88^. As shown in Fig. 8d, the proposed method achieved a score of 72.5, indicating good analytical performance.

## Conclusion

This approach offers a robust and efficient isocratic RP-HPLC method with satisfactory system suitability that was established and optimized for the concurrent quantification of favipiravir, sofosbuvir, ledipasvir, and remdesivir. Employing QbD principles through BBD enabled systematic exploration of the method’s key factors and facilitated the identification of optimal chromatographic conditions. The proposed method was evaluated for greenness, blueness, and whiteness to ensure its overall sustainability. The developed method offers a helpful analytical tool for quality control applications, particularly in pharmaceutical environments handling a wide range of antiviral agents. Its simplicity, cost-effectiveness, and reproducibility make it highly suitable for routine analysis and therapeutic monitoring in the context of pandemic response and preparedness.

## Data Availability

All data generated or analyzed during this study are included in this published article.
